# Effects of dance on cognitive function among older adults: a protocol for systematic review and meta-analysis

**DOI:** 10.1186/s13643-018-0689-6

**Published:** 2018-01-27

**Authors:** ASM Borhan, Patricia Hewston, Dafna Merom, Courtney Kennedy, George Ioannidis, Nancy Santesso, Pasqualina Santaguida, Lehana Thabane, Alexandra Papaioannou

**Affiliations:** 1 Department of Health Research Methods, Evidence, and Impact (HEI), McMaster University, Hamilton, ON Canada; 2 Geriatric Education and Research in Aging Sciences (GERAS) Centre, McMaster University, 88 Mapplewood Avenue, Hamilton, ON L8M 1W9 Canada; 3 Department of Medicine, McMaster University, Hamilton, ON Canada; 4 School of Science and Health, Western Sydney University, Penrith, Australia; 5 Biostatistics Unit, St Joseph Healthcare, Hamilton, ON Canada

## Abstract

**Background:**

Cognitive impairment is characterized by problems in thinking, memory, language, and judgment that are greater than cognitive changes in normal aging. Considering the unprecedented growth of the older adult population and the projected increase in the prevalence of cognitive impairment, it is imperative to find effective strategies to improve or maintain cognitive function in older adults. The objective of this review is to summarize the effects of dance versus any other control group on cognitive function, physical function, adverse events, and quality of life in older adults.

**Method:**

We will search the following databases MEDLINE, EMBASE, and Cochrane Central Register of Controlled Trials (CENTRAL) to identify the randomized controlled trials (RCTs) evaluating the effects of dance on cognitive function among older adults. Also, we will search http://apps.who.int/trialsearch, clinicaltrials.gov and conference abstracts to identify ongoing and unpublished studies. There will be no restrictions on language, date, or journal of publication. Reviewers will independently and in duplicate screen for eligible studies using pre-defined criteria. Data extraction from eligible studies will be performed independently and in duplicate. The Cochrane risk of bias tool will be used to assess the risk of bias of studies. Our primary outcome of interest is cognitive function, more specifically the executive function domain. We will include other domains as well such as processing speed and reaction time. Secondary outcomes of interest are physical function. The secondary outcomes also include adverse events including falls and quality of life. We will use Review Manager (RevMan 5.3) to pool the effect of dance for each outcome where possible. Results will be presented as relative risks along with 95% confidence intervals for dichotomous outcomes and as mean differences, or standardized mean differences along with 95% confidence intervals, for continuous outcomes. We will assess the certainty of the evidence using the GRADE approach and present findings in a Summary of Findings table.

**Discussion:**

This systematic review, to our best knowledge the first-ever, will synthesize the available evidence on the effects of dance on cognitive function among older people.

**Systematic review registration:**

PROSPERO CRD42017057138

**Electronic supplementary material:**

The online version of this article (10.1186/s13643-018-0689-6) contains supplementary material, which is available to authorized users.

## Background

There is unprecedented growth in the global population of older adults, aged 65 and over, with an increase from 8.5% in 2015 to 16.7% in 2050 [1]. Cognitive impairment is characterized by problems in thinking, memory, language, and judgment that are greater than cognitive changes in normal aging, which varies from mild to severe [2]. Decline in cognitive function has emerged as one of the major health challenges as one in two people over age 85 diagnosed with dementia while the estimated prevalence of mild cognitive impairment, among people aged 60 and over, is 5–36% in the year 2015 [3, 4]. Therefore, we need to identify interventions that can help older people to maintain physical and cognitive functions to allow for involvement in social activities and maintain independence [5]. Numerous studies have shown that aerobic exercise [6–10], muscle strengthening [8, 10], and coordinative activity [7, 11] improve cognitive ability.

However, physical activities vary considerably in the degree of sensorimotor complexity, cognitive demand, and degree of social interaction, and thus, the attenuation of cognitive decline may be dependent on the type of exercise [12]. Dance may be an effective intervention that synergistically imrpoves both cognitive and physical functions. Dance consists of complex elements, such as synchronization of movement to music, memorization of step sequence, and social interaction which, on its own, is recognized as having a beneficial effect on cognition [13]. It requires involvements of several cognitive and physical functions through perception, execution, memory, and motor skills [14].

Over the last decade, dance is gaining popularity as a therapeutic activity for improving the cognitive ability of older people, for example, dance therapy for Parkinson’s [15, 16] and dementia [17, 18]. A number of non-randomized [19, 20] and randomized trial [12, 21] studies have shown the effectiveness of dance on cognitive function. To our knowledge, this proposed systematic review is the first aimed to synthesize the evidence from randomized controlled trials measuring the effects of dance on cognitive function in older adults.

## Objectives

The objective of this systematic review is to determine the effects of dance on domains of cognitive function, physical function, adverse events, and quality of life among older adults. This review will address the following research question:

Among older adults (aged 55 or over), what are the effects of dance compared to any control group (physical activity, non-physical activity, or no activity), on cognitive function (primary), physical function (secondary), adverse events (secondary), and quality of life (secondary)?

## Methods and design

The guidelines of the Preferred Reporting Items for Systematic Reviews and Meta-Analysis Protocol (PRISMA-P) [22, 23] were used to prepare the protocol of this review (see Additional file 1 for the checklist). This protocol was registered on the PROSPERO International Prospective Register of Systematic Reviews (CRD42017057138).

### Data sources and search strategy

The electronic search strategy for Ovid MEDLINE (see Additional file 2 for the proposed search strategy) was developed with the assistance of an experienced information specialist. The following electronic databases will be searched since the earliest available data: MEDLINE, EMBASE, and Cochrane Central Register of Controlled Trials (CENTRAL). There will be no restrictions on language, date, or journal of publication. A combination of MeSH terms and keywords will be applied. Unpublished and ongoing trials will be identified on the International Clinical Trials Registry Platform (http://apps.who.int/trialsearch) and http://clinicaltrials.gov. In addition, we will search the conference abstract archives on the websites of the International Association of Gerontology and Geriatrics (IAGG), International Federation of Ageing (IFA), American Geriatrics Society (AGS), Gerontological Society of America, and Canadian Geriatrics Society (CGS) for all available abstracts present at all conferences until the search date. Finally, the reference lists of all relevant studies and review articles will be searched for studies not identified by electronic searches.

### Study eligibility

This review will include randomized controlled trials (RCTs) (using individual or cluster randomization) evaluating the effects of dance on cognitive function among older people. Non-randomized studies including cohort (retrospective or prospective), case-control, and case series will be excluded. No a priori restrictions on methodological quality will be imposed.

### Population

Participants aged 55 years or older will be included in this review. There will be no restrictions on the type of living arrangement (i.e., community-dwelling, nursing, or retirement residence).

### Intervention

Studies evaluating the effect of dance, e.g., social dance, ballroom, Latin, folk, salsa, comparative, tango, waltz, jazz, and creative dance, on cognitive function will be included. There will be no restrictions on the frequency or the duration of the dance intervention. Dance can be performed under the supervision of trained professionals or by participants themselves.

### Comparator

Studies for the effect of dance on cognitive function will be included if they compare dance to any control group (e.g., education, walking, tai chi, no structured activity). This review will exclude pharmacological agents as comparator.

### Outcome and measurement

The primary outcome of interest for this review is cognitive function, more specifically the executive function domain, such as task switching and response inhibition.

We will consider other domains such as processing speed, reaction time, verbal and visuospatial learning ability, working memory, and immediate and delayed memory.

The secondary outcome of interest is physical function, in particular the domain balance of physical function. Further, we will include other domains of physical function including walking speed. We also assess the effect of dance on adverse events, specifically falls, and quality of life.

Different tools may be used to measure these outcomes. We will consider all the tools used to measure these outcomes in the included studies.

### Screening and data extraction

Studies identified will be entered into the reference manager software EndNote X8 for screening. Two reviewers will independently and in duplicate screen relevant studies against the eligibility criteria. Inter-rater reliability in the application of eligibility criteria will be performed. Similarly, two reviewers will independently and in duplicate assess the full text of any article selected in the title or abstract screening process using the developed prescribed form for inclusion. Any disagreement will be resolved independently by a third reviewer. A PRISMA flow diagram [24] will be used to document the study selection process.

Data from the included studies will be extracted independently and in duplicate by two reviewers. A standardized data extraction form will be developed and piloted to ensure capture of all relevant data. Data related to study design and setting, participant demographics (e.g., age, gender, living status, comorbidity), description of interventions (e.g., dance type, frequency, session duration), and description of the comparator (e.g., comparator type, frequency, duration) as well as data on outcomes of interest will be collected.

Data extractors will discuss any disagreements, and where necessary, discrepancies will be resolved by a third reviewer.

### Study risk of bias assessment

The risk of bias of each study will be independently and in duplicate assessed by two reviewers. For any disagreement, a third reviewer will confirm the final assessment. Study authors will be contacted in the event of insufficient details to confidently assess the risk of bias.

The Cochrane risk of bias tool will be used to assess the risk of bias of included RCTs [25]. The assessments will be on the following domains: (1) random sequence generation, (2) allocation concealment, (3) blinding of participants and personnel, (4) blinding of outcome assessment, (5) incomplete outcome data, (6) selective reporting, and (7) other bias. For each domain, the risk of bias will be assessed as “low,” “high,” or “unclear.” An unclear risk will be assigned for a domain if an insufficient detail is reported and cannot be obtained from study authors. After the assignment of risk of bias, studies will be classified according to the following categories:

1. Low risk: studies with all domains at low risk of bias
2. High risk: studies with one or more domains at high risk of bias

### Statistical analysis

All study data will be entered into a Review Manager software (RevMan5.3) [26] to synthesize the evidence. Study characteristics will be summarized using frequencies (%) for categorical variables and mean (SD) or median (IQR) for continuous variables. Data on primary and secondary outcomes will be analyzed in aggregate using a random-effects model. Binary outcomes will be reported as risk ratios along with 95% confidence intervals. For continuous outcomes, pooled mean difference or standardized mean difference along with 95% confidence intervals will be reported. Missing means (standard deviations) will be approximated using medians (interquartile ranges) according to Hozo et al. [27], and approximate standard deviations will be calculated from interquartile ranges [25]. If necessary, skewed data will be log-transformed. Meta-analysis will be conducted using the software RevMan version 5.3. Furthermore, forest plots will be generated.

If it is not possible to perform meta-analysis for any outcome due to insufficient data, a qualitative synthesis will be performed. The unit of analysis will be limited to, when possible, an individual participant in each trial arm. In the case of multi-arm studies (e.g., several dance types compared to control), the combination of groups to create a single pair-wise comparison will be attempted, as recommended by the Cochrane Handbook for Systematic Reviews of Interventions [25]. In the case of cluster randomized trials, data will be adjusted for design effect to convert these into a trial on individual participants [25].

In the case of missing data, study authors will be contacted and a maximum of three attempts will be made to obtain the data. If data remain unavailable, a narrative description of these studies will be provided, and the potential impact of such missing data will be addressed in the “Discussion” section of the manuscript.

The degree of statistical heterogeneity will be evaluated from forest plots, using chi-square tests and the *I*^2^ statistic (*I*^2^ > 50% indicates moderate to substantial heterogeneity). Publication bias will be assessed using a funnel plot of all included studies (≥ 10). For continuous outcomes, the Egger test [28] will be used to detect funnel plot asymmetry while arcsine test [29] will be used for dichotomous outcomes.

### Subgroup and sensitivity analyses

The following additional subgroup analyses will be performed subject to the availability of sufficient data:

- Severity of cognitive impairment (e.g., dementia, mild cognitive impairment, subjective cognitive impairment). We hypothesize that people with dementia may improve cognitive ability more than those with less severe/mild cognitive impairment.
- Dance type (e.g., standing [line dancing; partnered dancing], seating [performed sitting in a chair]). We hypothesize that people who participate in standing dance will gain more cognitive ability than people who participate in seating dance.
- Study design: individual versus cluster randomization

We will conduct sensitivity analyses for the primary outcomes excluding studies with high risk of bias. Post hoc sensitivity analysis will be performed, when appropriate. For example, we will perform sensitivity analysis using different methods of imputation for studies with missing data.

### Certainty of the evidence

We will assess the certainty of the evidence for each outcome using the Grading of Recommendations Assessment, Development, and Evaluation (GRADE) approach [30]. The GRADE domains include risk of bias, inconsistency, indirectness, imprecision, and publication bias. We will produce a Summary of Findings table.

## Dissemination

The findings of this review will be submitted for publication in a peer-reviewed journal. In addition, the findings will be presented at local and national forums to reach out to clinicians and researchers of geriatrics, kinesiology, and gerontology community.

## Discussion

Cognitive function is a significant health concern among older people. To our best knowledge, our review will be the first to evaluate the evidence for the effects of dance on cognitive function among older adults. Our planned and broad search strategy will include published and unpublished studies. Our proposed systematic review and meta-analysis will synthesize the available evidence using rigorous methods, which will shed light on the effects of dance on cognitive function and permit identification of evidence gaps, therefore informing clinical decision-making and guide future research initiatives.

## Additional files

Additional file 1: PRISMA-P 2015 Checklist. This file contains Preferred Reporting Items for Systematic Review and Meta-analysis Protocols (PRISMA-P) 2015 checklist. (DOCX 30 kb)

Additional file 2: Proposed search strategy (or strategies) and terms. This file contains proposed search strategy for MEDLINE in OVID interface. (DOCX 12 kb)

## Abbreviations

AB: ASM Borhan
AP: Alexandra Papaioannou
CK: Courtney Kennedy
DM: Dafna Merom
GI: George Ioannidis
LT: Lehana Thabane
NS: Nancy Santesso
PH: Patricia Hewston
PS: Pasqualina Santaguida
