# Supplementary material for: Effects of dance on cognitive function among older adults: a protocol for systematic review and meta-analysis
Source: Syst Rev. 2018 Jan 27;7:24. doi: 10.1186/s13643-018-0689-6 (PMC5787313; doi:10.1186/s13643-018-0689-6)
Supplement: Supplementary file 2 — Proposed search strategy (or strategies) and terms. This file contains proposed search strategy for MEDLINE in OVID interface. (DOCX 12 kb) [file 13643_2018_689_MOESM2_ESM.docx]

**Proposed search strategy (or strategies) and terms**

Search strategy for MEDLINE using OVID interface

--------------------------------------------------------------------------------

1 danc*.mp.

2 tango.mp.

3 waltz.mp.

4 jazz.mp.

5 ballroom.mp.

6 polka.mp.

7 foxtrot.mp.

8 chacha.mp.

9 rumba.mp.

10 samba.mp.

11 bolero.mp.

12 salsa.mp.

13 1 or 2 or 3 or 4 or 5 or 6 or 7 or 8 or 9 or 10 or 11 or 12

14 exp Randomized Controlled Trial/

15 exp Controlled Clinical Trial/

16 randomized.ab.

17 randomized.ti.

18 placebo.ab.

19 placebo.ti.

20 drug therapy.fs.

21 randomly.ab.

22 randomly.ti.

23 trial.ab.

24 trial.ti.

25 groups.ab.

26 groups.ti.

27 clinical trials.mp.

28 14 or 15 or 16 or 17 or 18 or 19 or 20 or 21 or 22 or 23 or 24 or 25 or 26 or

27

29 Animals/

30 Humans/

31 29 not 30

32 28 not 31

33 13 and 32
